# The emerging threat of pre-extensively drug-resistant tuberculosis in West Africa: preparing for large-scale tuberculosis research and drug resistance surveillance

**DOI:** 10.1186/s12916-016-0704-5

**Published:** 2016-11-03

**Authors:** Florian Gehre, Jacob Otu, Lindsay Kendall, Audrey Forson, Awewura Kwara, Samuel Kudzawu, Aderemi O. Kehinde, Oludele Adebiyi, Kayode Salako, Ignatius Baldeh, Aisha Jallow, Mamadou Jallow, Anoumou Dagnra, Kodjo Dissé, Essosimna A. Kadanga, Emmanuel Oni Idigbe, Catherine Onubogu, Nneka Onyejepu, Aissatou Gaye-Diallo, Awa Ba-Diallo, Paulo Rabna, Morto Mane, Moumine Sanogo, Bassirou Diarra, Zingue Dezemon, Adama Sanou, Madikay Senghore, Brenda A. Kwambana-Adams, Edward Demba, Tutty Faal-Jawara, Samrat Kumar, Leopold D. Tientcheu, Adama Jallow, Samba Ceesay, Ifedayo Adetifa, Assan Jaye, Mark J. Pallen, Umberto D’Alessandro, Beate Kampmann, Richard A. Adegbola, Souleymane Mboup, Tumani Corrah, Bouke C. de Jong, Martin Antonio

**Affiliations:** 1 Vaccines and Immunity Theme, Medical Research Council Unit The Gambia, Banjul, The Gambia; 2 University of Ghana Medical School, Accra, Ghana; 3 Korle-Bu Teaching Hospital, Accra, Ghana; 4 Warren Alpert Medical School of Brown University, Providence, RI USA; 5 The Miriam Hospital, Providence, RI USA; 6 College of Medicine, University of Ibadan, Ibadan, Nigeria; 7 University College Hospital, Ibadan, Oyo 23402 Nigeria; 8 National Public Health Laboratory Services, Banjul, The Gambia; 9 Laboratoire National de Reference Mycobacteria, Lome, Togo; 10 Nigerian Institute of Medical Research, Lagos, Nigeria; 11 Laboratoire Bactériologie Virologie Aristide Le Dantec Sénégal, Dakar, Senegal; 12 National Institute of Public Health, Bissau, Guinea-Bissau; 13 SEREFO Program, University of Sciences, Techniques and Technologies of Bamako, Bamako, Mali; 14 Centre Muraz and the National TB Program, Ouagadougou, Burkina Faso; 15 Microbiology and Infection Unit, Warwick Medical School, University of Warwick, Coventry, UK; 16 Faculty of Infectious and Tropical Diseases, London School of Hygiene and Tropical Medicine, London, UK; 17 Department of Infectious Diseases Epidemiology, London School of Hygiene and Tropical Medicine, London, UK; 18 Disease Control and Elimination, Medical Research Council Unit, Serrekunda, The Gambia; 19 Institute of Tropical Medicine, Antwerp, Belgium; 20 Department of Paediatrics, Imperial College London, London, UK; 21 GSK Vaccines, Wavre, Belgium; 22 Department of Biochemistry, Faculty of Science, University of Yaoundé 1, Yaoundé, Cameroon; 23 National Tuberculosis/Leprosy Control Program, Banjul, The Gambia; 24 Health Services, Ministry of Health and Social Welfare, Banjul, The Gambia

**Keywords:** Tuberculosis, Extensively drug-resistant tuberculosis, West Africa, Drug-resistance surveillance, Capacity building

## Abstract

**Background:**

Drug-resistant tuberculosis (TB) is a global public health problem. Adequate management requires baseline drug-resistance prevalence data. In West Africa, due to a poor laboratory infrastructure and inadequate capacity, such data are scarce. Therefore, the true extent of drug-resistant TB was hitherto undetermined. In 2008, a new research network, the West African Network of Excellence for Tuberculosis, AIDS and Malaria (WANETAM), was founded, comprising nine study sites from eight West African countries (Burkina Faso, The Gambia, Ghana, Guinea-Bissau, Mali, Nigeria, Senegal and Togo). The goal was to establish Good Clinical Laboratory Practice (GCLP) principles and build capacity in standardised smear microscopy and mycobacterial culture across partnering laboratories to generate the first comprehensive West African drug-resistance data.

**Methods:**

Following GCLP and laboratory training sessions, TB isolates were collected at sentinel referral sites between 2009–2013 and tested for first- and second-line drug resistance.

**Results:**

From the analysis of 974 isolates, an unexpectedly high prevalence of multi-drug-resistant (MDR) strains was found in new (6 %) and retreatment patients (35 %) across all sentinel sites, with the highest prevalence amongst retreatment patients in Bamako, Mali (59 %) and the two Nigerian sites in Ibadan and Lagos (39 % and 66 %). In Lagos, MDR is already spreading actively amongst 32 % of new patients. Pre-extensively drug-resistant (pre-XDR) isolates are present in all sites, with Ghana showing the highest proportion (35 % of MDR). In Ghana and Togo, pre-XDR isolates are circulating amongst new patients.

**Conclusions:**

West African drug-resistance prevalence poses a previously underestimated, yet serious public health threat, and our estimates obtained differ significantly from previous World Health Organisation (WHO) estimates. Therefore, our data are reshaping current concepts and are essential in informing WHO and public health strategists to implement urgently needed surveillance and control interventions in West Africa.

## Background

**Fig. 1:**
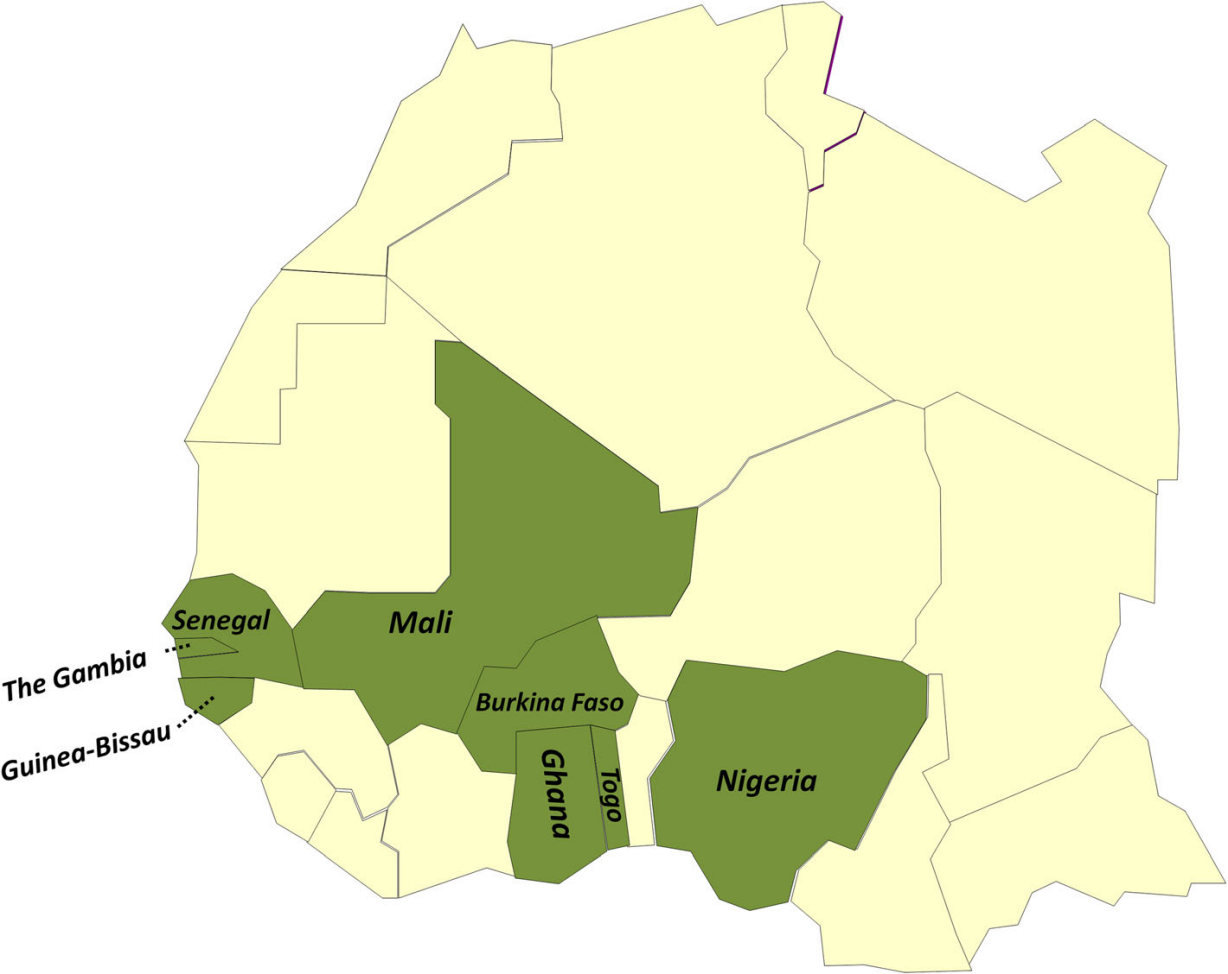
Participating WANETAM sites. Coordinated by the Medical Research. Council Unit (*MRC*), The Gambia, the following partner sites were part of the capacity building activities and drug resistance surveys: (1) Senegal, Laboratoire Bactériologie Virologie, Le Dantec, Dakar; (2) The Gambia, National Public Health Laboratory Services, Banjul; (3) Guinea-Bissau, National Institute of Public Health (*INASA*), Bissau; (4) Mali, SEREFO (HIV/TB Research and Training Center) FMOS, University of STT, Bamako; (5) Burkina Faso, Centre Muraz and the National TB Program (*NTP*), Ouagadougou; (6) Ghana, Korle Bu Teaching Hospital, Accra; (7) Togo, Laboratoire National de Référence (*LNR*) des Mycobactéries, Lome; (8) Nigeria, Nigerian Institute of Medical Research (*NIMR*), Lagos; (9) Nigeria, College of Medicine, University of Ibadan

West Africa comprises 15 countries and is home to 245 million inhabitants who are highly affected by communicable diseases. A limited sub-regional health and laboratory infrastructure leaves the region especially vulnerable not just to major infectious diseases, such as tuberculosis (TB), malaria, meningitis and human immunodeficiency virus (HIV), but also to devastating emerging epidemics, including the largest ever Ebola outbreak [1]. Creating laboratory capacity and fostering regional collaborations within West Africa will allow countries to respond with rapid concerted action to emerging public health threats and to conduct clinical trials to address local health needs and inform global health policies. To close knowledge gaps, leading scientists and research institutes from the sub-region joined forces to establish the West African Network of Excellence for TB, AIDS and Malaria (WANETAM) [2]. Funded by the European and Developing Countries Clinical Trials Partnership (EDCTP), WANETAM’s mission from 2008–2014 has been to build capacity to train members in standardised laboratory techniques essential to prepare the region for clinical trials and public health relevant diagnostics and research [2]. The network is the first of its kind and comprises nine sentinel sites from eight West African countries: one Portuguese-speaking, four French-speaking and three English-speaking countries (see Fig. 1).

Within the TB work package, spearheaded by the Medical Research Council (MRC) Unit, The Gambia, emphasis was on assessing the distribution of drug-resistant *Mycobacterium tuberculosis* in West Africa, as routine TB surveillance mechanisms and data on TB resistance are scarce in the region [3]. Detecting multi-drug-resistant tuberculosis (MDR-TB), defined as *Mycobacterium tuberculosis* resistant to rifampicin and isoniazid, is increasingly important, as drug resistance has emerged as one of the biggest challenges for TB control [4]. Furthermore, it is crucial to understand whether prevalent MDR isolates in the region will be susceptible to second-line drugs or have already acquired resistance to the quinolones or injectable aminoglycosides (pre-extensively drug-resistant [pre-XDR]) or resistance to both classes (extensively drug-resistant [XDR]), as these phenotypes are associated with a worse prognosis [4]. Besides the comparably low success rates of curing patients infected with MDR-TB (48 %) [4], in most West African countries either adequate MDR therapy is not readily available or sub-optimal treatment regimens are in use. If we can show that drug-resistant TB is important in WANETAM countries, this will justify implementation of standardised (inter)national drug susceptibility testing (DST) surveillance systems and roll-out of adequate MDR treatment programs across West Africa. In addition, an international TB drug resistance study prepares the region for future multi-centre clinical TB trials of new regimens for MDR-TB.

## Methods

### Training activities

The MRC Unit, The Gambia, was a named WANETAM Node of Excellence (NoE) and became the focal point for coordinating Good Clinical Laboratory Practice (GCLP) and all TB-related activities. In order to prepare the WANETAM network for a multi-centre study (and future clinical trials), common standards between the study sites had to be implemented. This was achieved by conducting a series of initial workshops (see Table 1).

**Table 1:** Capacity building and training activities within WANETAM

Year	Workshop title	Content	Duration	Location	Number of trainees
2010	Good Clinical Laboratory Practice (GCLP)	Principles of GCLP	1 week	MRC	18
2011	Basic Mycobacteriology	Microscopy	4 weeks	MRC	18
		Isolation of mycobacteria			
		Solid and liquid culture			
		Storage of isolates			
2013	IATA Certification Training	Shipment of isolates	2 days	Benin	23
2012	Molecular Epidemiology of MDR-TB in West Africa	Boiled lysate/genomic DNA extraction	2 weeks	MRC	18
		Polymerase chain reaction (PCR)			
		Hain MTBDR*plus*			
		Spoligotyping			
2013	Rapid Detection, Rapid Action: TB diagnosis with GeneXpert	Background on RT-PCR testing	1 week	MRC	18
		GeneXpert technology and maintenance			
		Practical Xpert MTB/RIF			
		Result interpretation			
2013	From Bench to Genome, a Simplified Perspective	Bioinformatics course	1 day	MRC	18
2014	Data Analysis and Publishing	Statistics	1 week	MRC	18
		Critical appraisal of data			
		Writing skills			
2010–2014	12 Visits to Partner Sites	Customised training	8 weeks	WANETAM sites	–

*MTBDRplus* assay for rapid detection of multi-drug-resistant TB, *Xpert MTB/RIF* assay for rapid detection of TB and resistance to rifampicin

In parallel, laboratories of partner institutes were initially assessed in their proficiency towards performing routine diagnostics, including smear microscopy, culture and isolate storage. To assist the member countries in their efforts to implement the current state-of-the-art diagnostics, several regional workshops, during which participants received not only theoretical knowledge but also hands-on practice in laboratory activities, were conducted (see Table 1). To guarantee comparability of diagnostic techniques between study sites, all assays were performed according to disseminated Standard Operating Procedures (SOPs) and GCLP. The initial workshops conveyed basic diagnostic methods, such as microscopy, solid/liquid culture and storage of isolates, which provided essential knowledge needed for conducting drug resistance surveys. To reinforce the knowledge gained and to troubleshoot arising problems, there were 12 customised on-site visits (consisting of 8 weeks in total) of MRC staff to partner laboratories. Additional training included a variety of DNA extraction protocols and advanced molecular methods such as phenotypic DST testing or the Hain GenoType MTBDR*plus* and GenXpert MTB/RIF for genotypic DST testing. Training in a basic genotyping method (spoligotyping) was provided for early detection of potential outbreaks of resistant clones. For an overview of the conducted workshops and their detailed content, see Table 1.

### Sample collection and shipment

We collected consecutive TB isolates from new and retreatment patients (according to WHO case definitions [3]) from nine West African partner institutes (see Fig. 1) in the following eight West African countries: Burkina Faso, The Gambia, Ghana, Guinea-Bissau, Mali, Nigeria, Senegal and Togo. The isolates were collected between 2009–2013 from the TB referring centres in each capital city, and for Togo these represented countrywide samples. Nigeria was the only country with two sites, one in Lagos and the other in Ibadan. Conducting a standardised sampling approach was challenging, as the catchment areas of recruited patients between the partner sites varied. For example, the Nigerian Institute of Medical Research (NIMR) in Lagos is a countrywide referral centre for patients with suspected drug-resistant TB, whereas in The Gambia all of the patients were recruited from the Greater Banjul Area. All isolates were processed according to common SOPs and shipped to MRC fully compliant with the International Air Transport Association (IATA) Dangerous Goods Regulations (https://www.iata.org/services/Microsites/DGR/en/index.html) [5]. Resistance profiles to first- and second-line drugs were performed at MRC Unit, The Gambia, and isolates were archived in a biobank.

### First- and second-line drug susceptibility testing

Drug susceptibility testing (DST) was performed to determine the resistance pattern of isolates to first- and second-line anti-TB drugs. The standard protocol for DST for the first-line drugs streptomycin (STR, 1 μg/mL), isoniazid (INH, 0.1 μg/mL), rifampicin (RIF, 1 μg/mL) and ethambutol (EMB, 5 μg/mL) in MGIT 960 (Becton Dickinson, Oxford Science Park, Oxford, UK) was followed according to the manufacturer’s instructions [6]. Phenotypic DST for second-line drugs was performed on identified MDR isolates in MGIT 960 (Becton Dickinson, Oxford Science Park, Oxford, UK) using kanamycin (KAN, 2.5 μg/mL) capreomycin (CAP, 2.5 μg/mL), ofloxacin (OFX, 2 μg/mL) and ethionamide (ETH, 5 μg/mL) (Sigma-Aldrich, St. Louis, Mo, USA) [7].

### Quality assurance

To assure accuracy of DST results, the MRC TB Diagnostics Laboratory participates in external quality assurance from the National External Quality Assessment Service (NEQAS), UK (http://www.ukneqas.org.uk/). A blinded panel provided by WHO through the National Mycobacterium Reference Laboratory in the UK (https://www.gov.uk/government/collections/national-mycobacterium-reference-laboratory-nmrl) was analysed for quality assurance of speciation and first- and second-line DST. In addition, a standardised susceptible H37Rv (ATCC 27249) laboratory strain was included in each batch. The MRC Diagnostics laboratories’ first- and second-line DST together with other diagnostic assays achieved ISO15189:2012 accreditation in July 2015.

### Data management and statistical analysis

Sample data were entered in real time at MRC Unit, The Gambia, into a custom-built Structured Query Language (SQL) database with an Access front end. Data were checked thoroughly for consistency before the database was locked in June 2015. The extracted data were analysed using Stata/SE v12.1 (2011, Stata Statistical Software: Release 12, StataCorp LP, College Station, TX, USA). Categorical data were summarised using appropriate descriptive count and percentage statistics. To achieve appropriate coverage, confidence intervals were constructed using the Wilson interval. All analyses were split by site and treatment history status (new and retreatment).

## Results

### Drug resistance in West Africa

#### Mycobacterial collection and patient characteristics

**Fig. 2:**
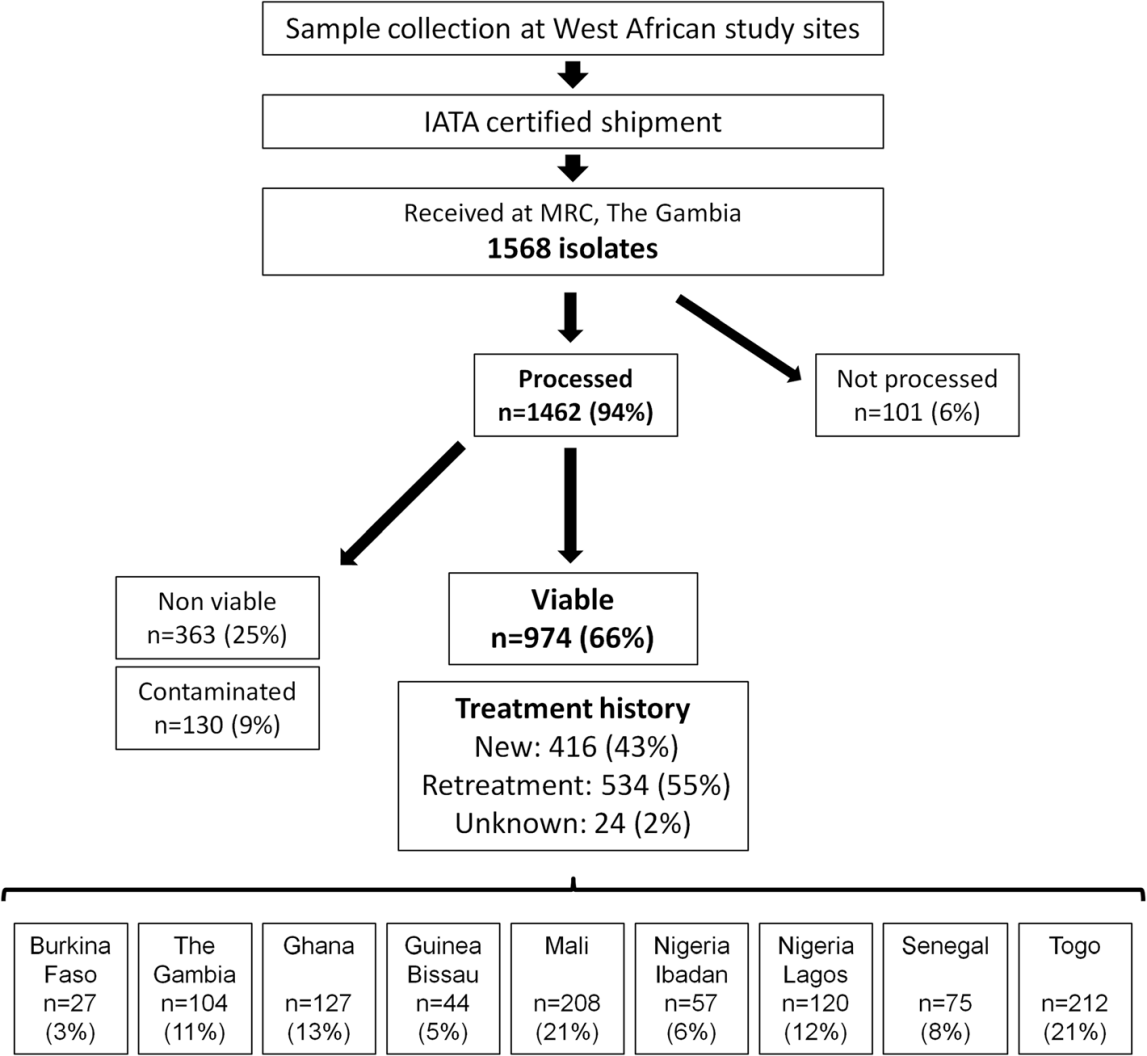
Flowchart of collected, shipped and processed samples included in the present study

Isolates were collected from new and retreatment patients from each of the study sites, analysed and then archived for future research in a newly founded biobank at MRC Unit, The Gambia. A total of 1568 isolates were collected across the nine study sites and sent to MRC Unit, The Gambia. Out of 1462 that were processed, 9 % were contaminated and 25 % were non-viable following the shipment. In total, 974 isolates (66 %) were included in the drug resistance survey and stored at –70 °C (see Fig. 2). For an overview of patient demographics, including treatment history, sex, age and HIV status, see Table 2.

**Table 2:** Patient characteristics of study population at individual WANETAM study sites

		Burkina Faso		The Gambia		Ghana		Guinea-Bissau		Mali		Nigeria Ibadan		Nigeria Lagos		Senegal		Togo		All study sites	
		*n*	%	*n*	%	*n*	%	*n*	%	*n*	%	*n*	%	*n*	%	*n*	%	*n*	%	*n*	%
Total		27	(3)	104	(11)	127	(13)	44	(5)	208	(21)	57	(6)	120	(12)	75	(8)	212	(21)	974	(100)
Treatment history	New	20	(74)	9	(9)	15	(12)	32	(73)	150	(72)	13	(23)	28	(23)	55	(73)	94	(44)	416	(43)
	Retreatment	1	(4)	95	(91)	112	(88)	4	(9)	58	(28)	44	(77)	88	(73)	20	(27)	112	(53)	534	(55)
	Unknown	6	(22)	0	(0)	0	(0)	8	(18)	0	(0)	0	(0)	4	(3)	0	(0)	6	(3)	24	(2)
Age	0–14	0	(0)	0	(0)	1	(1)	1	(3)	4	(2)	3	(5)	1	(1)	4	(5)	3	(1)	17	(2)
	15–24	3	(14)	31	(30)	16	(13)	10	(26)	50	(24)	6	(10)	16	(14)	12	(16)	27	(13)	171	(18)
	25–34	3	(14)	31	(30)	35	(29)	12	(31)	78	(38)	21	(37)	45	(39)	25	(34)	57	(27)	307	(31)
	35–44	8	(38)	20	(20)	32	(26)	7	(18)	38	(18)	15	(26)	32	(28)	14	(19)	57	(27)	223	(23)
	45–54	3	(14)	10	(10)	26	(21)	6	(15)	21	(10)	8	(14)	12	(10)	7	(9)	42	(20)	135	(14)
	55–64	2	(10)	7	(7)	4	(3)	1	(3)	11	(5)	2	(4)	9	(8)	5	(7)	13	(6)	54	(5)
	65+	2	(10)	3	(3)	8	(7)	2	(5)	6	(3)	2	(4)	1	(1)	7	(9)	9	(4)	40	(4)
	Unknown	6	–	2	-	5	-	5	–	0	–	0	–	4	–	1	–	4	–	27	(3)
Sex	Female	7	(33)	31	(30)	29	(24)	18	(44)	51	(25)	23	(40)	54	(46)	17	(23)	76	(36)	306	(31)
	Male	14	(67)	73	(70)	93	(76)	23	(56)	157	(75)	34	(60)	63	(54)	58	(77)	136	(64)	650	(67)
	Unknown	6	–	0	-	5	-	3	–	0	–	0	–	3	–	0	–	1	–	18	(2)
HIV	Negative	0	(0)	56	(54)	25	(20)	0	(0)	172	(83)	42	(74)	46	(38)	15	(20)	115	(54)	471	(48)
	Positive	0	(0)	5	(5)	3	(2)	12	(27)	29	(14)	4	(7)	27	(23)	12	(16)	36	(17)	128	(14)
	Unknown	27	(100)	43	(41)	99	(78)	32	(73)	7	(3)	11	(19)	47	(39)	48	(64)	61	(29)	375	(38)

#### Resistance to first-line drugs and MDR

**Fig. 3:**
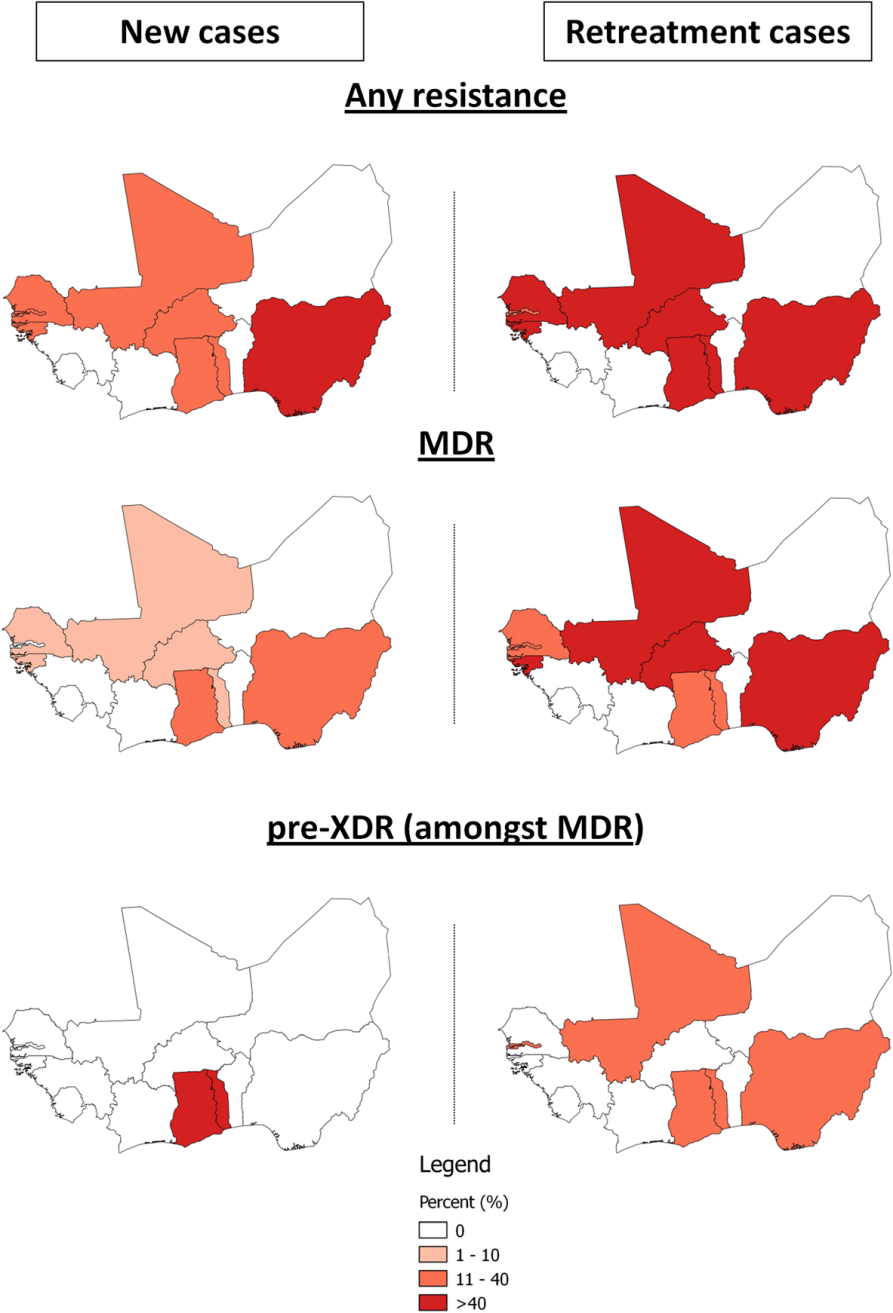
Geographical distribution and prevalence of drug-resistant *M. tuberculosis* complex isolates in WANETAM study sites. The proportions of resistant strains within the total bacterial population per country are mapped (for Nigeria the average of the two study sites, Lagos and Ibadan, is displayed). The maps are stratified by new (*left column*) and retreatment patients (*right column*). The *upper panel* shows geographical distribution of the proportion of isolates with any first-line drug resistance. The *centre panel* shows the proportion of MDR amongst the total bacterial population. The *lower panel* shows the proportional geographical distribution of pre-XDR within the total population of MDR isolates. The sample sizes for each country are as follows (country name [*n* = new patients/*n* = retreatment patients]): Burkina Faso [20/1], The Gambia [9/95], Ghana [15/112], Guinea-Bissau [32/4], Mali [150/58], Nigeria [41/132] (Lagos and Ibadan combined), Senegal [55/20], Togo [94/112] (for further details see Tables 3 and 4)

In total, 39 % of all isolates were resistant to at least one first-line drug, and MDR isolates were found at all sites (Table 3 and Fig. 3). Consistent with previous studies, we found that bacteria isolated from retreatment patients were more than four times more likely to be resistant to one or more first-line drugs when compared to mycobacteria from new patients (OR 4.4 [95 % CI 3.3–5.9]).

**Table 3:** First-line drug resistance by study site and treatment history

Study site	Treatment history		First-line drug resistance				Total	DST profiles of resistant isolates											
			Pan susceptible		Any resistance			RMP mono		INH mono		SM mono		EMB mono		PDR		MDR	
			*N*	%	*n*	%		*N*	%	*n*	%	*n*	%	*n*	%	*N*	%	*n*	%
Burkina Faso	All		18	(67)	9	(33)	27	0	(0)	2	(7)	1	(4)	0	(0)	4	(15)	2	(7)
		New	13	(65)	7	(35)	20	0	(0)	2	(10)	1	(5)	0	(0)	3	(15)	1	(5)
		Retreatment	0	(0)	1	(100)	1	0	(0)	0	(0)	0	(0)	0	(0)	0	(0)	1	(100)
		Unknown	5	(83)	1	(17)	6	0	(0)	0	(0)	0	(0)	0	(0)	1	(17)	0	(0)
The Gambia	All		80	(77)	24	(23)	104	1	(1)	4	(4)	6	(6)	0	(0)	1	(1)	12	(11)
		New	7	(78)	2	(22)	9	0	(0)	1	(11)	1	(11)	0	(0)	0	(0)	0	(0)
		Retreatment	73	(77)	22	(23)	95	1	(1)	3	(3)	5	(5)	0	(0)	1	(1)	12	(13)
Ghana	All		64	(50)	63	(50)	127	1	(0)	7	(5)	13	(10)	0	(0)	9	(7)	33	(26)
		New	11	(73)	4	(27)	15	0	(0)	1	(7)	1	(7)	0	(0)	0	(0)	2	(13)
		Retreatment	53	(47)	59	(53)	112	1	(1)	6	(5)	12	(11)	0	(0)	9	(8)	31	(28)
Guinea-Bissau	All		34	(77)	10	(23)	44	2	(4)	1	(2)	1	(2)	1	(2)	2	(5)	3	(7)
		New	24	(75)	8	(25)	32	2	(6)	1	(3)	1	(3)	1	(3)	2	(6)	1	(3)
		Retreatment	2	(50)	2	(50)	4	0	(0)	0	(0)	0	(0)	0	(0)	0	(0)	2	(50)
		Unknown	8	(100)	0	(0)	8	0	(0)	0	(0)	0	(0)	0	(0)	0	(0)	0	(0)
Mali	All		143	(69)	65	(31)	208	1	(0)	7	(3)	7	(3)	0	(0)	11	(5)	39	(19)
		New	127	(85)	23	(15)	150	1	(1)	7	(5)	6	(4)	0	(0)	4	(3)	5	(3)
		Retreatment	16	(28)	42	(72)	58	0	(0)	0	(0)	1	(2)	0	(0)	7	(12)	34	(59)
Nigeria/Ibadan	All		31	(54)	26	(46)	57	1	(2)	1	(2)	4	(7)	0	(0)	3	(5)	17	(30)
		New	11	(85)	2	(15)	13	0	(0)	1	(8)	1	(8)	0	(0)	0	(0)	0	(0)
		Retreatment	20	(45)	24	(55)	44	1	(2)	0	(0)	3	(7)	0	(0)	3	(7)	17	(39)
Nigeria/Lagos	All		27	(22)	93	(78)	120	0	(0)	6	(5)	11	(9)	0	(0)	5	(4)	71	(59)
		New	11	(40)	17	(61)	28	0	(0)	1	(4)	5	(18)	0	(0)	2	(7)	9	(32)
		Retreatment	16	(18)	72	(82)	88	0	(0)	5	(6)	6	(7)	0	(0)	3	(3)	58	(66)
		Unknown	0	(0)	4	(100)	4	0	(0)	0	(0)	0	(0)	0	(0)	0	(0)	4	(100)
Senegal	All		60	(80)	15	(20)	75	0	(0)	3	(4)	1	(1)	0	(0)	3	(4)	8	(11)
		New	49	(90)	6	(11)	55	0	(0)	1	(2)	1	(2)	0	(0)	1	(2)	3	(5)
		Retreatment	11	(55)	9	(45)	20	0	(0)	2	(10)	0	(0)	0	(0)	2	(10)	5	(25)
Togo	All		142	(67)	70	(33)	212	4	(2)	14	(7)	16	(7)	1	(0)	5	(2)	30	(14)
		New	79	(84)	15	(16)	94	1	(1)	1	(1)	7	(7)	0	(0)	1	(1)	5	(5)
		Retreatment	62	(55)	50	(45)	112	3	(3)	13	(12)	8	(7)	1	(1)	3	(3)	22	(20)
		Unknown	1	(17)	5	(83)	6	0	(0)	0	(0)	1	(17)	0	(0)	1	(17)	3	(50)
All study sites	All		599	(61)	375	(39)	974	10	(1)	45	(5)	60	(6)	2	(0)	43	(4)	215	(22)
		New	332	(80)	84	(20)	416	4	(1)	16	(4)	24	(6)	1	(0)	13	(3)	26	(6)
		Retreatment	253	(47)	281	(53)	534	6	(1)	29	(5)	35	(7)	1	(0)	28	(5)	182	(34)
		Unknown	14	(58)	10	(42)	24	0	(0)	0	(0)	1	(4)	0	(0)	2	(8)	7	(29)

The overall proportion of resistance to any drug is displayed as well as a detailed description of resistant isolates and their resistant patterns, stratified by mono-resistance to rifampicin (*RMP*), isoniazid (*INH*), streptomycin (*SM*), ethambutol (*EMB*), poly-drug-resistance (*PDR*, resistance to a combination of any two first-line drugs except for MDR) and multi-drug-resistance (*MDR*, resistance to at least both RMP and INH)

Amongst retreatment patients, Mali (59 %) and the Nigerian study sites in Lagos (66 %) and Ibadan (39 %) had the highest percentages of MDR-TB. All other study sites had high numbers of MDR amongst retreatment patients with The Gambia at 13 % being the lowest. With the exception of The Gambia and Nigeria/Ibadan, the majority of countries identified MDR isolates from new patients, with Nigeria/Lagos (32 %) and Ghana (13 %) reporting the largest proportions (Table 3).

#### Resistance to second-line drugs and XDR/pre-XDR

Amongst all MDR strains tested for second-line drug resistance, no XDR was found (see Table 4). However 41 (21 %) pre-XDR strains resistant to OFX or KAN and/or CAP were found in 199 MDR isolates. Interestingly, only Ghana and Togo reported pre-XDR strains from new patients, whereas pre-XDR from retreatment patients was found in The Gambia, Ghana, Mali, Nigeria (Ibadan/Lagos) and Togo (see Table 4, Fig. 3).

**Table 4:** Second-line drug resistance by study site and treatment history

Study site	Treatment history		Second-line drug resistance amongst MDR				Total MDR with second-line DST done	Pre-XDR drug resistance profiles of MDR isolates							
			Susceptible		Pre-XDR			Fluoroquinolones		Injectables					
								OFX		KAN		CAP		KAN/CAP	
			*N*	%	*n*	%		*n*	%	*n*	%	*n*	%	*n*	%
Burkina Faso	All		1	(100)	0	(0)	1	0	(0)	0	(0)	0	(0)	0	(0)
		New	–	–	–	–	0	–	–	–	–	–	–	–	–
		Retreatment	1	(100)	0	(0)	1	0	(0)	0	(0)	0	(0)	0	(0)
The Gambia	All		9	(75)	3	(25)	12	0	(0)	0	(0)	2	(17)	1	(8)
		New	0	(0)	0	(0)	0	0	(0)	0	(0)	0	(0)	0	(0)
		Retreatment	9	(75)	3	(25)	12	0	(0)	0	(0)	2	(17)	1	(8)
Ghana	All		20	(61)	13	(39)	33	3	(9)	2	(6)	7	(21)	1	(3)
		New	0	(0)	2	(100)	2	0	(0)	2	(100)	0	(0)	0	(0)
		Retreatment	20	(65)	11	(35)	31	3	(10)	0	(0)	7	(23)	1	(3)
Guinea-Bissau	All		1	(100)	0	(0)	1	0	(0)	0	(0)	0	(0)	0	(0)
		New	1	(100)	0	(0)	1	0	(0)	0	(0)	0	(0)	0	(0)
		Retreatment	–	–	–	–	0	–	–	–	–	–	–	–	–
Mali	All		30	(79)	8	(21)	38	1	(3)	1	(3)	5	(13)	1	(3)
		New	5	(100)	0	(0)	5	0	(0)	0	(0)	0	(0)	0	(0)
		Retreatment	25	(76)	8	(24)	33	1	(3)	1	(3)	5	(16)	1	(3)
Nigeria/Ibadan	All		13	(76)	4	(23)	17	2	(12)	0	(0)	2	(12)	0	(0)
		New	0	(0)	0	(0)	0	0	(0)	0	(0)	0	(0)	0	(0)
		Retreatment	13	(76)	4	(23)	17	2	(12)	0	(0)	2	(12)	0	(0)
Nigeria/Lagos	All		59	(91)	6	(9)	65	6	(9)	0	(0)	0	(0)	0	(0)
		New	8	(100)	0	(0)	8	0	(0)	0	(0)	0	(0)	0	(0)
		Retreatment	51	(89)	6	(11)	57	6	(10)	0	(0)	0	(0)	0	(0)
Senegal	All		5	(100)	0	(0)	5	0	(0)	0	(0)	0	(0)	0	(0)
		New	2	(100)	0	(0)	2	0	(0)	0	(0)	0	(0)	0	(0)
		Retreatment	3	(100)	0	(0)	3	0	(0)	0	(0)	0	(0)	0	(0)
Togo	All		20	(74)	7	(26)	27	1	(4)	1	(4)	3	(11)	2	(7)
		New	2	(40)	3	(60)	5	1	(20)	0	(0)	1	(20)	1	(20)
		Retreatment	18	(82)	4	(18)	22	0	(0)	1	(4)	2	(9)	1	(4)
All study sites	All		158	(79)	41	(21)	199	12	(6)	4	(2)	19	(9)	6	(3)
		New	18	(78)	5	(22)	23	1	(4)	2	(9)	1	(4)	1	(4)
		Retreatment	140	(80)	36	(20)	176	11	(6)	2	(1)	18	(10)	5	(3)

No extensively drug-resistant isolates (*XDR*) were found in the survey. The proportion of pre-XDR within the population of MDR isolates is displayed as well as a detailed description of pre-XDR isolates and their resistance patterns, stratified by mono-resistance to ofloxacin (*OFX*), kanamycin (*KAN*) and capreomycin (*CAP*) and the combination of the latter two (*KAN/CAP*)

## Discussion

Due to scanty data, most of West Africa remains a ‘blank’ or is classified as ‘no data’ on drug resistance figures in WHO TB reports [3]. According to the WHO, lack of laboratory infrastructure is responsible for this shortcoming, and this urgently needs to be overcome [3]. A prerequisite for conducting TB drug surveys, and a necessity for any clinical TB trial, is the ability to successfully perform smear microscopy in combination with mycobacterial cultures, both of which were established at the West African study sites within the WANETAM network. In recently designed surveys, such as the one in Senegal, the primary screen for rifampicin resistance is based on the GeneXpert MTB/RIF, followed by culture and DST for rifampicin-resistant sputa and a sub-set of the sensitive ones. Following common SOPs, the nine WANETAM study sites collected sputa and isolated mycobacteria using solid or liquid cultures. Ultimately we described a comprehensive overview of drug resistance in West Africa for the first time.

We found a high proportion of isolates that were resistant to one or more first-line drugs (39 %) across the West African sites. In addition almost a quarter (22 %) of all isolates tested showed MDR phenotypes and are therefore unlikely to respond to first-line drug therapy or even to the standardised retreatment (category II) regimen that adds only streptomycin as the new drug to the first-line therapy. These infections require specific future TB control measures such as adequate diagnostics and availability of effective therapy based on DST results. Although high rates of MDR in retreatment patients are systematically found across all WANETAM study sites, the situation appears especially alarming in Nigeria (Lagos 66 %, Ibadan 39 %) and Mali (59 %). In Lagos, for instance, 32 % of all new patients with their first ever episode of TB were already presenting with widely circulating MDR isolates. Our findings support previous publications from Nigeria [8–12] and Burkina Faso [13, 14]; however, variations in the respective MDR prevalence estimates of these publications are considerable, due to differences in sampling strategy, collection time points and location, making an overall comparison difficult. Burkina Faso, Nigeria and Niger were the only WANETAM countries that previously reported XDR isolates [3, 15]. Although we did not identify any XDR isolates in our set of samples, six countries demonstrated the emergence of pre-XDR strains. Despite having the highest MDR prevalence, it was not the site in Lagos, Nigeria but Ghana that yielded the highest proportion of pre-XDR isolates in 11 % and 35 %, respectively, within their MDR population of new and retreatment patients. Overall, 21 % of all MDR strains were pre-XDR across all WANETAM sites combined. As second-line treatment of these strains will be impaired due to lack of susceptibility to either fluoroquinolones or injectable drugs, these pre-XDR bacteria are on the verge of developing the full XDR phenotype if no effective interventions are instituted, and they constitute a major public health threat in the region. Of further concern is that Ghana and Togo, independently from each other, identified pre-XDR amongst new patients. This suggests that these strains have started spreading within the general population of these two neighbouring West African countries, although confirmation by molecular fingerprinting methods of circulating isolates is needed.

**Fig. 4:**
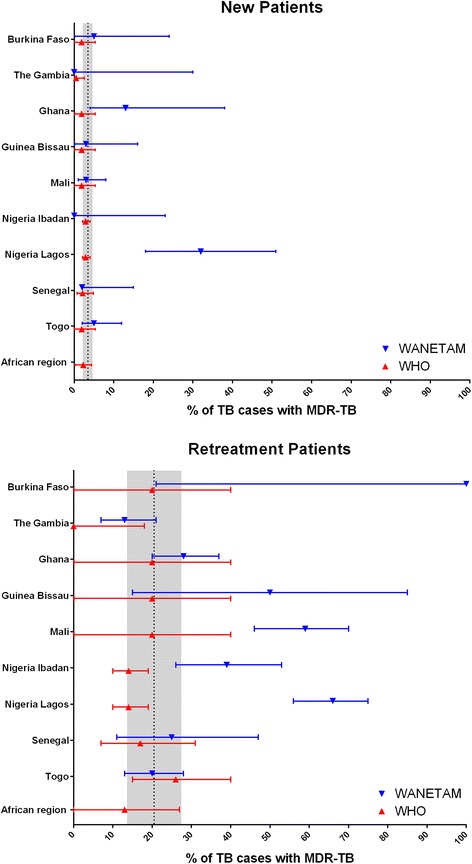
Comparison between WANETAM (*blue*) and WHO Global TB report, 2014 (*red*) MDR prevalence estimates. The country-specific WHO estimates were retrieved from the recent Global TB report, 2014, at http://www.who.int/tb/country/data/profiles/en/ (accessed 11.08.2015). The mean (*triangle*) percentage of MDR per all TB isolates is given for each country/study site and Africa, together with the respective 95 % CI. The *upper panel* shows results for new patients, the *lower panel* retreatment patients. The *shaded areas* display the global average (*dotted line*), including the range from the lower to upper limit of the 95 % CI. Note that in case of a potential strong selection bias, a limitation of the present study, the confidence intervals around the WANETAM mean might still reflect an overestimate of the true prevalence

To put the WANETAM results in context, we compared our estimates with the latest data from the WHO Global Tuberculosis Report 2014 (Fig. 4) [3]. To date only three WANETAM countries conducted previous drug-resistance surveys. While a Senegalese survey is presently on-going and Nigeria completed a survey in between 2009–2010 [16], data from The Gambia was collected more than a decade ago in 1999 [17]. As no previous data existed for several countries in 2013, WHO reports a common MDR estimate (new patient: 1.9 % [0.1 –5.3 %], retreatment: 20 % [0.1–40 %]) for Mali, Guinea-Bissau, Ghana and Burkina Faso.

Our results allow us to update and complement previous and/or missing data reported to WHO from these eight WANETAM countries (see Fig. 4). While our estimates are in concordance for Togo and Senegal, we found significantly higher MDR prevalence in retreatment patients in the Nigerian and Malian sites when compared with the WHO estimated data [3]. This is not surprising, especially in Nigeria, where a 2012 nationwide TB prevalence survey found WHO estimates were 50 % of the true TB burden [16]. In contrast to the WHO estimates, none of the confidence intervals from our nine study sites included zero (in retreatment patients), providing strong evidence that MDR isolates are truly prevalent at all sites (Fig. 4). WANETAM data tend to be higher than WHO prevalences, highlighting the possibility that drug resistance in West Africa is currently underestimated (see Fig. 4). For instance, amongst new and retreatment patients, respectively, five out of nine and seven out of nine WANETAM sites were above the global TB prevalence average, and seven out of nine and eight out of nine WANETAM sites were above the estimated African MDR prevalence average.

Our study has limitations. First of all, sample collection in Burkina Faso and Guinea-Bissau was limited, and therefore the sample sizes were relatively small. We accounted for that by displaying 95 % CI for all study sites wherever appropriate. Secondly, selection bias based on the ‘catchment’ populations of the participating sites is likely to contribute to the high resistance rates, especially at NIMR in Lagos, which included a referral population who had been identified as resistant elsewhere. Also, despite thorough training, we cannot exclude the potential misclassification of treatment history (new versus retreatment) or infer the missing treatment history data. As no HIV status data were available for the majority of the patients, we were not able to investigate the role of HIV co-infection on MDR rates in our study. Finally, we did not include amikacin in the second-line DST, as it was not sustainably available in West Africa at the time of the initiation of the WANETAM network in 2009.

The presented MDR data, together with the documented emerging spread of pre-XDR in Ghana and Togo, indicate that the drug-resistance problem in West Africa may be greater than currently assumed, highlighting the urgent need for countrywide drug resistance surveys according to WHO guidelines. While awaiting such robust and unbiased results, our data should already prompt the implementation of continuous surveillance of all retreatment patients in participating countries. Such a system is ideally based on molecular screens, such as with the GeneXpert MTB/RIF, followed by additional molecular and phenotypic testing at the National Reference Laboratories. Moreover, the increasing detection of patients with MDR-TB stresses the need for the wider availability of effective treatment. Such efforts are already on-going, such as the roll-out of the 9-month short-course MDR regimen [18] in West and Central African countries with support from the International Union Against Tuberculosis and Lung Disease (IUTLD), beyond Niger, Benin and Cameroon [19, 20], which were early adopters of this regimen and report high rates of treatment success. As demonstrated, the WANETAM network has established essential laboratory capacity to conduct future clinical TB trials. A major challenge, however, for any successful network is its capability to function sustainably and independently. Encouragingly, WANETAM’s capacity building efforts have already had several positive consequences for member states beyond the initially defined network activities. For instance, the National TB Program of The Gambia recently conducted the first Gambian TB prevalence study, The Gambian Survey of Tuberculosis Prevalence (GAMSTEP). Similarly, the Chest Clinic Laboratory at the Korle-Bu Teaching Hospital in Ghana, which did not perform culture in the past, was accredited as the country’s National TB Reference Laboratory in the course of the WANETAM project. SEREFO in Bamako was selected as the diagnostic laboratory during the Malian response to the Ebola epidemic in 2014. During the same outbreak, IATA certified shippers in multiple countries, trained by WANETAM, were often the only staff available to send clinical samples of Ebola patients to respective reference laboratories. Most encouragingly, Senegal, Mali and Benin have hosted their own regional workshops in which both WANETAM member and non-member states, such as Chad, Rwanda or Democratic Republic of Congo (amongst others), were trained in classical microbiological and molecular methods.

## Conclusion

WANETAM accomplished the first steps in producing regional TB research of public health relevance in West Africa. As the established TB network is entirely based on South-South collaborations, partners face both similar and unique challenges, and can reach out across the region for further training exchanges and research collaborations. WANETAM is a crucial stepping stone in moving West Africa forward towards independent and internationally competitive TB research, for both the individual institutes and for multi-centred TB trials across the whole sub-region.

One of the most important achievements of WANETAM so far is the finding that drug-resistant TB could become a serious public health problem in West Africa if required control measures are not taken. This is not only because of high drug-resistant TB rates amongst retreatment patients but especially amongst new patients, showing that on-going transmission is currently insufficiently controlled. As the problem of emerging drug resistance is multi-facetted and sub-regional, control strategies need to improve on various levels and between countries. Implementing required quality standards (SOPs and GCLP) and laboratory infrastructure, as done by WANETAM, can therefore only be a first step in the successful control of drug-resistant TB in West Africa. We hope that our data could serve as a foundation for common West African guidelines and policies, jointly developed by the individual National TB Programs (NTPs) under the guidance of the West African Health Organisation (WAHO). Those guidelines are needed to ultimately tackle the challenges the sub-region is facing. Ideally, scientific networks, such as WANETAM, should consequently be maintained as powerful platforms that would have the capacity to bring the various stakeholders together and facilitate such a process. Therefore, we encourage international donors, such as EDCTP or WHO, to provide further future funds to allow the creation of new and continuation of already existing scientific networks in Africa and the developing world.

## Abbreviations

CAP: Capreomycin
DST: Drug susceptibility testing
EDCTP: European and Developing Countries Clinical Trials Partnership
EMB: Ethambutol
ETH: Ethionamide
GCLP: Good Clinical Laboratory Practice
HIV: Human immunodeficiency virus
IATA: International Air Transport Association
INH: Isoniazid
MDR: Multi-drug-resistant
NEQAS: National External Quality Assessment Service
NoE: Node of Excellence
NTP: National TB Program
OFX: Ofloxacin
OR: Odds ratio
Pre-XDR: Pre-extensively drug-resistant
RIF: Rifampicin
SOP: Standard Operating Procedure
STR: Streptomycin
TB: Tuberculosis
WAHO: West African Health Organisation
WANETAM: West African Network of Excellence for Tuberculosis, AIDS and Malaria
WHO: World Health Organisation
XDR: Extensively drug-resistant

## References

[1] Roca A, Afolabi MO, Saidu Y, Kampmann B (2015). Ebola: a holistic approach is required to achieve effective management and control. J Allergy Clin Immunol..

[2] Miiro GM, Oukem-Boyer OO, Sarr O, Rahmani M, Ntoumi F (2013). EDCTP regional networks of excellence: initial merits for planned clinical trials in Africa. BMC Public Health..

[3] World Health Organization (2014). WHO report, global tuberculosis report.

[4] World Health Organization (2014). Multidrug-resistant tuberculosis (MDR-TB), 2014 update.

[5] International Air Transport Association (2013). Infectious substances shipping guidelines — The complete reference guide for pharmaceutical & health professionals.

[6] Ardito F, Posteraro B, Sanguinetti M, Zanetti S, Fadda G (2001). Evaluation of BACTEC Mycobacteria Growth Indicator Tube (MGIT 960) automated system for drug susceptibility testing of *Mycobacterium tuberculosis*. J Clin Microbiol..

[7] World Health Organization (2008). The Stop TB Department. Policy guidance on drug-susceptibility testing (DST) of second-line antituberculosis drugs.

[8] Ani AE, Idoko J, Dalyop YB, Pitmang SL (2009). Drug resistance profile of *Mycobacterium tuberculosis* isolates from pulmonary tuberculosis patients in Jos, Nigeria. Trans R Soc Trop Med Hyg..

[9] Daniel O, Osman E (2011). Prevalence and risk factors associated with drug resistant TB in South West, Nigeria. Asian Pac J Trop Med..

[10] Kehinde AO, Adebiyi EO (2013). Molecular diagnosis of MDR-TB using GenoType MTBDRplus 96 assay in Ibadan, Nigeria. Niger J Physiol Sci..

[11] Lawson L, Yassin MA, Abdurrahman ST, Parry CM, Dacombe R (2011). Resistance to first-line tuberculosis drugs in three cities of Nigeria. Trop Med Int Health..

[12] Otu A, Umoh V, Habib A, Ansa V (2014). Prevalence and clinical predictors of drug-resistant tuberculosis in three clinical settings in Calabar, Nigeria. Clin Respir J..

[13] Sangare L, Diande S, Badoum G, Dingtoumda B, Traore AS (2010). Anti-tuberculosis drug resistance in new and previously treated pulmonary tuberculosis cases in Burkina Faso. Int J Tuberc Lung Dis..

[14] Sangare L, Diande S, Kouanda S, Dingtoumda BI, Mourfou A (2010). *Mycobacterium tuberculosis* drug-resistance in previously treated patients in Ouagadougou, Burkina Faso. Ann Afr Med..

[15] Saleri N, Badoum G, Ouedraogo M, Dembele SM, Nacanabo R (2010). Extensively drug-resistant tuberculosis, Burkina Faso. Emerg Infect Dis..

[16] Sebastian V. From facility to finding drug resistance mutations: drug resistance survey in Nigeria. 45th Union World Conference on Lung Health. Barcelona; 2014. p. 57.

[17] Adegbola RA, Hill P, Baldeh I, Otu J, Sarr R (2003). Surveillance of drug-resistant *Mycobacterium tuberculosis* in The Gambia. Int J Tuberc Lung Dis..

[18] Van Deun A, Maug AK, Salim MA, Das PK, Sarker MR (2010). Short, highly effective, and inexpensive standardized treatment of multidrug-resistant tuberculosis. Am J Respir Crit Care Med..

[19] Kuaban C, Noeske J, Rieder HL, Ait-Khaled N, Abena Foe JL (2015). High effectiveness of a 12-month regimen for MDR-TB patients in Cameroon. Int J Tuberc Lung Dis..

[20] Piubello A, Harouna SH, Souleymane MB, Boukary I, Morou S (2014). High cure rate with standardised short-course multidrug-resistant tuberculosis treatment in Niger: no relapses. Int J Tuberc Lung Dis..

